# Cerebral Hyperperfusion Syndrome Presenting as Epilepsia partialis Continua Following Carotid Stenting: A Case Report

**DOI:** 10.7759/cureus.50015

**Published:** 2023-12-05

**Authors:** Saleh M Alkhonezan, Souda El Sheikh, Hanan K Aljaidi, Naif Faisal Almutairi

**Affiliations:** 1 Neurology Department, Prince Sultan Military Medical City, Riyadh, SAU

**Keywords:** cas, seizure, cerebral hyperperfusion syndrome, carotid artery stenting, carotid stenosis

## Abstract

Cerebral hyperperfusion syndrome (CHS) is a rare prodrome of symptoms, including headaches, focal neurological deficits, seizures, or encephalopathy. Herein, we report a unique case of focal motor status epilepticus (Epilepsia partialis continua [EPC]). A 76-year-old male underwent right carotid stenting (CAS) for symptomatic high-grade stenosis. Immediately post-operation, he was recovering well without neurologic deficits; however, four days later, his blood pressure increased, and he experienced focal motor seizures involving the left arm and face without impaired awareness. He was managed with antihypertensive and antiseizure medications. Subsequently, his respiratory function worsened, necessitating intubation for status epilepticus. Repeated imaging demonstrated only the previously known infarcts without cerebral edema, bilaterally patent carotid arteries or any signs of acute infarct or intracerebral hemorrhage.

While CHS is a rare syndrome with well-documented symptomatology, focal motor status epilepticus can occur abruptly without the more typical CHS symptoms, despite the best preventive measures.

## Introduction

Cerebral hyperperfusion syndrome (CHS) is a rare complication encountered in the treatment of long-standing severe carotid stenosis using carotid endarterectomy (CEA) or carotid artery stenting (CAS) [1]. The incidence rate of CHS is higher following CEA (1.9%) than after CAS (1.16%) [2]. The mechanism behind CHS is presumed to be a failure of normal autoregulation of cerebral blood flow [2]. Patients who develop CHS can present with seizures, hypertension, unilateral headaches, or localized neurological impairments [3,4]. Epilepsia partialis continua (EPC) is an atypical clinical condition that manifests as recurrent and sometimes intractable, focal-onset seizures associated with retained awareness [5]. It is an uncommon presentation, even in patients who develop CHS following CEA or CAS. This case report aims to highlight the importance of identifying EPC as a clinical manifestation of CHS to ensure that proper attention is paid and adequate care is delivered to affected patients.

## Case presentation

Patient information

A 76-year-old male patient presented with a history of hypertension (off medication for one month) and two previous ischemic strokes without a residual deficit. He had significant stenosis of the bilateral internal carotid arteries (ICAs) (right ICA: almost 75%; left ICA: 90%). He had been receiving dual antiplatelet and hypolipemic therapy. The patient underwent elective cerebral angioplasty and stenting of the right ICA. He was discharged two days later with no evidence of a neurologic deficit and a well-controlled blood pressure ranging from 122/77-135/80 mmHg. Four days after stenting, he developed sudden abnormal movements and was readmitted to our hospital. He was experiencing focal seizures characterized by gaze deviation, head turning to the left side, jerky movements of the left arm, and intact awareness.

Clinical findings

At the time of admission, the patient was conscious enough to follow commands. There were no other signs of lateralizing or focal neurological deficits from a stroke evaluation conducted using the National Institutes of Health Stroke Scale (NIHSS: 0).

Diagnostic assessment

**Figure 1:**
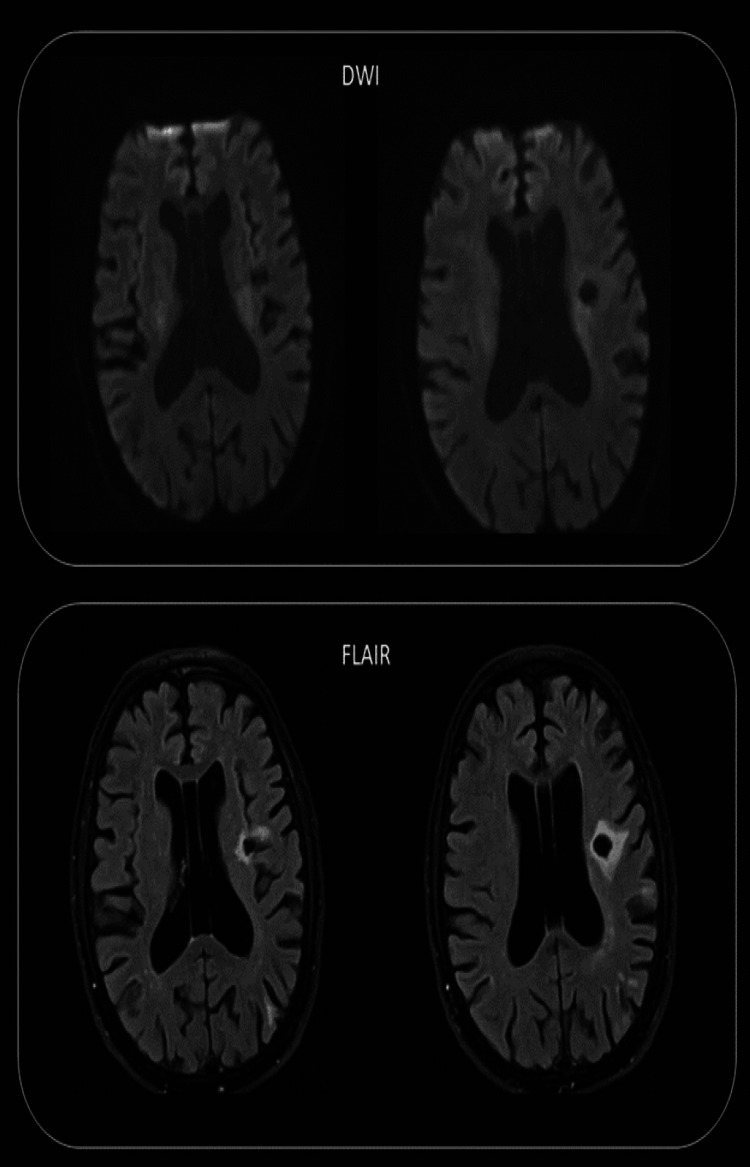
MRI brain diffusion-weighted imaging (DWI) and fluid-attenuated inversion recovery (FLAIR). Pre-stenting, demonstration of chronic lacunar infarctions.

**Figure 2:**
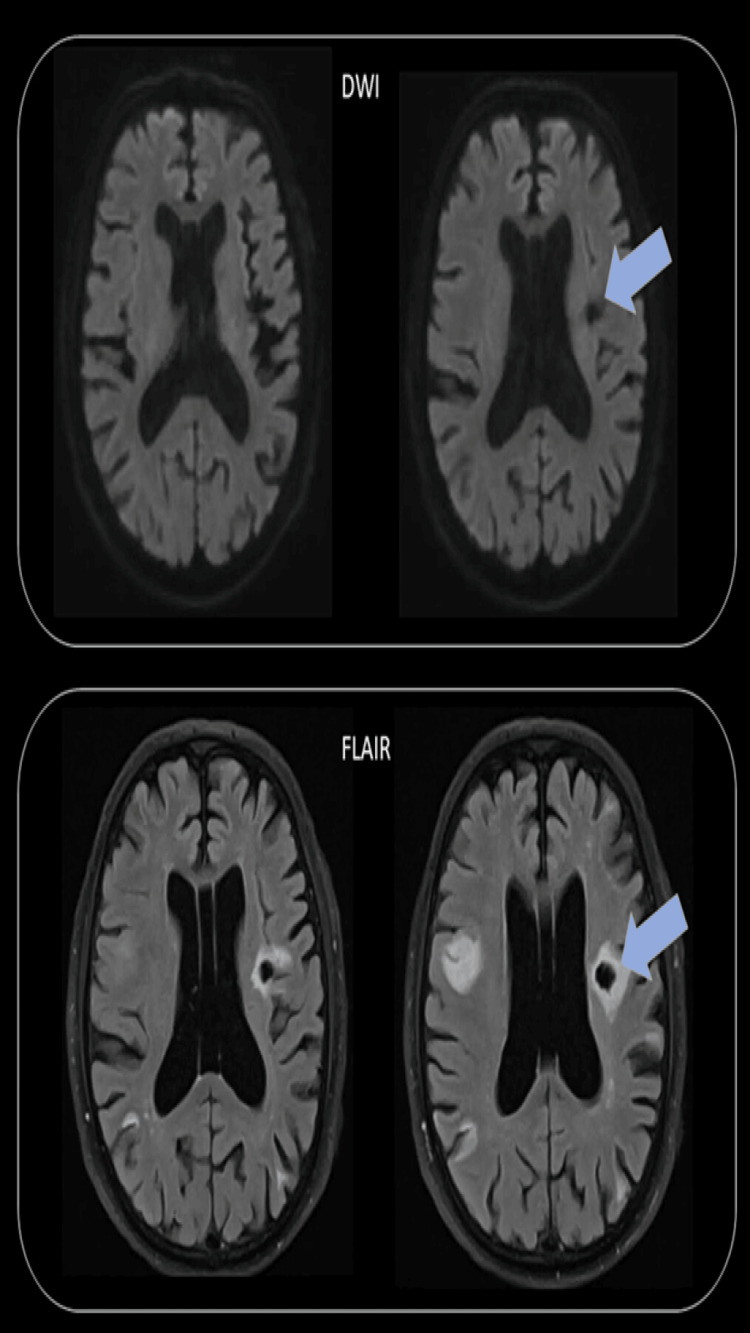
MRI brain diffusion-weighted imaging (DWI) and fluid-attenuated inversion recovery (FLAIR). Post-stenting. (Arrow) demonstration of chronic lacunar infarctions.

The patient’s laboratory results, including all hematological and biochemical tests, were unremarkable. Neither computed tomography (CT) nor magnetic resonance imaging (MRI) of the brain showed any signs of acute ischemic stroke, and only redemonstration of old strokes was observed (Figures 1, 2). Lectroencephalography (EEG) showed frequent repetitive rhythmic discharges from the right hemisphere in the form of rhythmic theta/delta activity lasting up to 40 seconds.

Therapeutic intervention

Despite multiple doses of midazolam and a loading dose of levetiracetam, the patient continued to have focal motor seizures without a decline in respiratory status. Accordingly, we decided to keep him on 500 mg of Levetiracetam twice a day and added 600 mg of Oxcarbazepine twice a day. His seizure persisted for another two days; hence, the levetiracetam dose was increased to 1000 mg twice a day and 100 mg of Lacosamide twice a day was added. After that, his seizure stopped.

Follow-up and outcomes

The patient was discharged and continued on 1000 mg of Levetiracetam twice a day, 600 mg of Oxcarbazepine twice a day, and 100 mg of Lacosamide twice a day. Six months after discharge, the patient’s seizures were well controlled, with no relapses.

## Discussion

Herein, we report a rare case of the development of a potentially refractory seizure as a consequence of ICA recanalization. EPC is a rare neurological condition characterized by predominantly motor partial seizures involving continuous or repetitive jerky (mostly clonic/myoclonic) movements of a particular region of the body [5]. These seizures may be prolonged, lasting from minutes to days [5]. A few studies have described cases of EPC in patients with encephalitis, metabolic derangement (e.g., hyperuricemia, uremia, hypo/hyperglycemia, hyponatremia), neoplastic processes (e.g., meningioma, oligodendroglioma), or stroke [5]. However, EPC as a clinical manifestation of CHS is extremely rare. To the best of our knowledge, there is only one published report of refractory focal seizure in a 76-year-old male patient with hypertension, a history of a previous stroke, and 99% stenosis in the left ICA [6]. The patient underwent a successful left ICA endarterectomy; however, he developed refractory focal status epilepticus the next day. He did not respond to any of the following treatments: Midazolam, Lorazepam, intubation and sedation with Propofol, or high doses of anti-seizure medications, including Lacosamide, Phenytoin, Levetiracetam, and Midazolam infusion. Unfortunately, the patient succumbed on postoperative day 15. Despite having a similar clinical profile (an elderly male developing refractory focal status epilepticus associated with CHS post carotid stenting that continued for two days), our patient showed some response to Midazolam, Levetiracetam, and Oxcarbazepine. The addition of Lacosamide on the following day improved his response, and his seizures subsequently ended.

## Conclusions

EPC is a neurological emergency that may be infrequently [1] induced as a consequence of ICA recanalization. To our knowledge, this is only the second instance of a patient developing focal status epilepticus due to CHS after (CAS). The existing literature has linked cerebral hyperperfusion to multiple risk factors, such as recent ipsilateral stroke, coronary artery disease, and uncontrolled blood pressure. However, none of these risk factors were present in our patient. Our patient was diagnosed with EPC associated with CHS based on clinical findings and was treated with levetiracetam, oxcarbazepine, and lacosamide. The patient showed significant improvement, with no relapses to date. This case report emphasizes the importance of paying attention to the abrupt onset of focal motor seizures and subsequent refractory focal status epilepticus in patients undergoing (CAS) or endarterectomy for carotid artery stenosis. Furthermore, this case highlights the fact that CHS incidents can occur abruptly, appearing as isolated focal motor seizures without the more typical CHS symptoms, despite the best preventive measures.
